# The survival impact of radiotherapy on synchronous metastatic rectal cancer: metastatic site can serve for radiotherapy-decision

**DOI:** 10.7150/jca.70894

**Published:** 2022-04-04

**Authors:** Yuan Zhou, Dan Wang, Fengbo Tan, Zhongyi Zhou, Lilan Zhao, Cenap Güngör, Qian Pei, Yuqiang Li, Wenxue Liu

**Affiliations:** 1 Department of General Surgery, Xiangya Hospital, Central South University, Changsha, China.; 2 National Clinical Research Center for Geriatric Disorders, Xiangya Hospital, Central South University, Changsha, China.; 3 Department of General Visceral and Thoracic Surgery, University Medical Center Hamburg-Eppendorf, Hamburg, Germany.; 4 Department of Thoracic surgery, Fujian Provincial Hospital, Fuzhou, China.; 5 Department of Cardiology, Xiangya Hospital, Central South University, Changsha, China.

**Keywords:** rectal cancer, radiotherapy, metastatic site, overall survival, SEER database

## Abstract

**Purpose:** The metastatic site seems to represent a malignancy with a different biological characteristic. Radiotherapy, as a successful, well-tolerated, cost-effective and time-efficient intervention, is able to provide clear benefits for the treatment of locally advanced rectal cancer and has become an essential component of palliative oncology care. The real-world effect of radiotherapy on the survival outcomes of metastatic rectal cancer (mRC) patients might do exist and was worth exploring.

**Patients and methods:** Data were extracted from the Surveillance, Epidemiology, and End Results (SEER) database in this retrospective analysis. The statistical methods included Pearson's chi-square test, Log-rank test, Cox regression model and propensity score matching (PSM).

**Results:** The multivariable Cox regression displayed that radiotherapy may not be used as a prognostic factor for mRC (p=0.057). However, radiotherapy may be associated with the prognosis if the metastatic site was excluded from the multivariate analysis (p<0.001). Radiotherapy seemed to fail to improve OS before PSM (p<0.001) and after PSM without the metastatic site as a matching factor (p<0.001). Nevertheless, there was no significant survival difference between radiotherapy and non-radiotherapy cohort after PSM with the metastatic site as a matching factor (p=0.057). All of M1a rectal cancer patients appear to obtain survival benefit from radiotherapy without the impact of PSM (p<0.001). Notwithstanding, radiotherapy was associated with improved OS of patients with rectal liver-limited metastasis (p=0.023) and did not appear to provide survival benefit for rectal lung-limited (p=0.386) and other-limited metastasis (p=0.385). Both of M1b mRC with and without liver metastasis did not seem to obtain survival benefit from radiotherapy.

**Conclusions:** Carefully selected data from the SEER database suggested that radiotherapy appears to improve overall survival only in patients with rectal liver-limited metastasis.

## Introduction

Colorectal cancer (CRC) is ranked as the top third malignancy in males and the second in females 1, and includes approximately 30%-50% rectal cancer (RC) 2. Metastasis is considered as the main cause of high mortality among rectal cancer patients 3. About 15-20% of RC exhibited distant metastasis at the time of diagnosis 4. Currently, the advancements in diagnostics, surgical techniques, new oncologic drugs and radiotherapy have significantly improved prognosis of rectal cancer, including prolonged survival outcomes of metastatic rectal cancer (mRC) 5.

Several previous studies reported that the metastatic site is an important prognostic factor for synchronous metastatic colorectal cancer 6, 7. More important, the metastatic site seems to represent a malignancy with a different biological characteristics 7. However, many studies analyzed metastatic rectal cancer as a whole without considering the metastatic site 8, 9, which may provide an inaccurate conclusion. Radiotherapy, as a successful, well-tolerated, cost-effective and time-efficient intervention, is able to provide clear benefits for the treatment of stage II/III rectal cancer and has become an essential component of palliative oncology care 8, 10, 11. However, it is not yet clear about the effect of radiotherapy on survival in the treatment of mRC. The real-world effect of radiotherapy on the survival outcomes of mRC patients might do exist and was worth exploring.

This study herein took advantage of the large patient population of the Surveillance, Epidemiology, and End Results (SEER) database to comprehensively examine the impact of radiotherapy on survival outcomes of mRC based on the metastatic site. These data can inform rectal oncologists in counseling patients with stage IV rectal cancer with synchronous metastatic disease seeking prognostic information when weighing radiotherapy decisions.

## Material and methods

### Patients Screening

**Figure 1:**
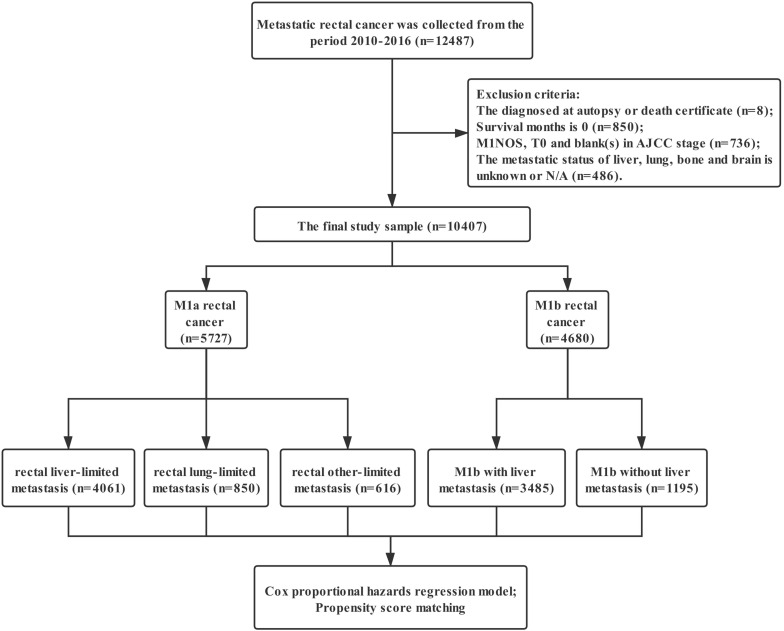
The flow diagram.

Data were extracted from the SEER linked database in this retrospective analysis. The SEER Program of the National Cancer Institute is an authoritative source of information on cancer incidence and survival in the United States (U.S.) that is updated annually. The rectal adenocarcinoma patients (ICD-O-3: 8140, 8144, 8145, 8201, 8210, 8211, 8213, 8220, 8221, 8253, 8255, 8260, 8261, 8262, 8263, 8310, 8323, 8480, 8481, 8490) with distant metastasis was collected from the period 2010-2016, 12,487 patients in total. Exclusion criteria: the diagnosed at autopsy or death certificate (n=8); Survival months is 0 (n=850); M1NOS, T0 and blank(s) in AJCC stage (n=736); the metastatic status of liver, lung, bone and brain is unknown or N/A (n=486); the final study sample contained 10,407 patients (Figure 1).

For each patient, the following data was acquired: insurance, age at diagnosis, marital status, gender, race, grade, histological type, T stage, N stage, regional nodes examined (RNE), CEA, surgery for primary tumor, metastatic site, radiotherapy and chemotherapy. We defined surgery with RNE ≥12 as standard proctectomy and that with RNE <12/NOS as simplified proctectomy. The definition of rectal liver-limited and lung-limited metastasis is M1a rectal cancer with liver and lung metastases at the time of diagnosis. Rectal other-limited metastasis included M1a rectal cancer with bone, brain and unknown site metastasis.

### Statistical Analysis

Intergroup comparisons were analyzed using Pearson's chi-square test. Log-rank test was used to compare overall survival (OS) between different groups. A hazard ratio (HR) and a 95% confidence interval (CI) were evaluated by a univariate and multivariate Cox proportional hazards regression model. Univariate analysis of variables with significant differences was included in the Cox regression model for multivariate analysis. In order to eliminate the influence of other variables, we conducted a 1:1 propensity score matching (PSM). Statistical analyses were performed with IBM SPSS statistics trial ver. 25.0 (IBM, Armonk, NY, USA). All reported p-values lower than 0.05 were considered significant.

## Results

### Patient Characteristics

The characteristics of patients with metastatic rectal cancer enrolled from the SEER database were summarized in Table 1. The total population included 5727 cases (55.03%) of M1a rectal cancer (liver-limited: 4061, 39.02%; lung-limited: 850; other-limited: 816, 7.84%) and 4680 patients (44.97%) with M1b rectal cancer (with liver metastasis: 3485, 33.49%; without liver metastasis: 1195, 11.48%). 3540 patients (34.02%) with metastatic rectal cancer received radiotherapy and non-radiotherapy group contained 6867 cases (65.98%) in this study. Metastatic rectal cancer patients with T3-4 and N+ tend to receive radiotherapy. Interestingly, there was no significant difference regarding metastatic site between radiotherapy and non-radiotherapy group (p=0.129). However, the proportion of patients with rectal liver-limited metastasis receiving radiotherapy (1301/4061, 32.04%) was lower than that of those with rectal lung-limited (390/850, 45.88%) and other-limited metastasis (464/816, 56.86%), and the rate of M1b rectal cancer patients with liver metastasis (944/3485, 27.09%) was also lower than that of those without liver metastasis (441/1195, 36.90%).

### The effect of radiotherapy on the total population

**Figure 2:**
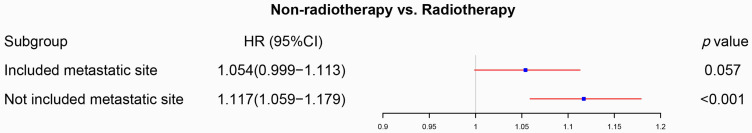
The forest plot was used to display the role of radiotherapy in the multivariable Cox regression. Radiotherapy cannot be used as a prognostic factor for mRC (p=0.057), but became an important prognostic factor if the metastatic site was excluded from the multivariate analysis (p<0.001). (The results were extracted from Table S1.)

**Figure 3:**
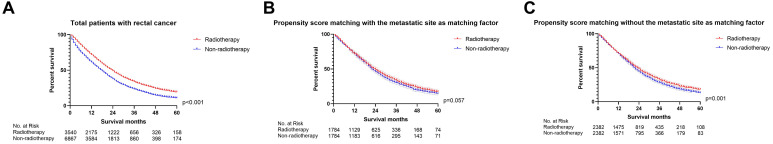
The survival curves showed that **(A)** radiotherapy was able to improve OS before PSM (p<0.001); **(B)** there was no significant survival difference between radiotherapy and non-radiotherapy cohort after PSM with the metastatic site as a matching factor (p=0.057); **(C)** radiotherapy can improve OS after PSM without the metastatic site as a matching factor (p<0.001). (The results of PSM were summarized in Table S2.)

We firstly applied univariable and multivariable Cox regression analysis to explore the effect of radiotherapy on metastatic rectal cancer patients (Table S1). Radiotherapy, age, marital status, race, pathologic grade, histologic type, T staging, N staging, surgery, chemotherapy, CEA and metastatic site were significant for overall survival in the univariable Cox regression model and brought into multivariable analysis. The multivariable Cox regression displayed that radiotherapy cannot be used as a prognostic factor for mRC (p=0.057, Figure 2). However, radiotherapy became an important prognostic factor if the metastatic site was excluded from the multivariate analysis (p<0.001, Figure 2). The metastatic site seems to be an important factor affecting the sensitivity of mRC to radiotherapy. Then PSM was utilized to verify the results of the multivariable Cox regression analysis. Characteristics of mRC patients after PSM with or without the metastatic site as a matching factor were showed in Table S2. Radiotherapy was able to improve OS before PSM (p<0.001, Figure 3A) and after PSM without the metastatic site as a matching factor (p<0.001, Figure 3C). However, there was no significant survival difference between radiotherapy and non-radiotherapy cohort after PSM with the metastatic site as a matching factor (p=0.057, Figure 3B). Therefore, we decided to analyze the effect of radiotherapy on mRC based on metastatic site.

### The effect of radiotherapy on M1a metastatic rectal cancer

**Figure 4:**
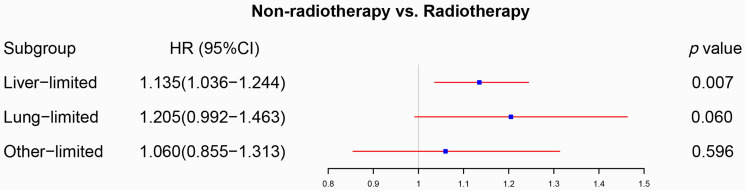
The forest plot displayed the effect of radiotherapy on M1a rectal cancer. Radiotherapy can be used as a prognostic factor for rectal liver-limited metastasis (p=0.007) but failed to improve survival for rectal lung-limited (p=0.060) and other-limited metastasis (p=0.596). (The results were extracted from Table S3.)

**Figure 5:**
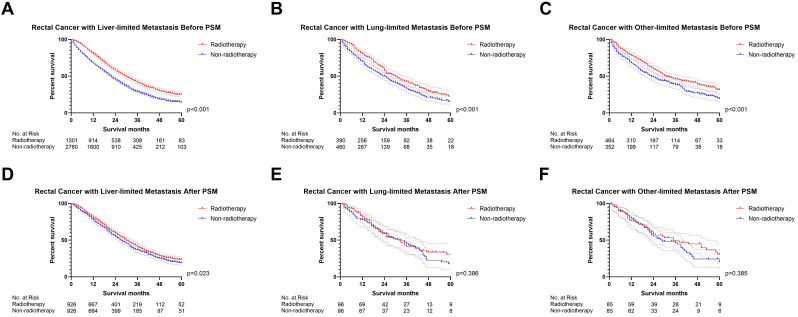
The survival curves demonstrated that** (A)** rectal liver-limited metastasis **(B)** rectal lung-limited metastasis and** (C)** rectal other-limited metastasis can obtain survival benefit from radiotherapy before PSM (p<0.001); **(D)** radiotherapy was able to improve OS of patients with rectal liver-limited metastasis (p=0.023) after PSM; **(E)** radiotherapy cannot provide survival benefit for rectal lung-limited (p=0.386) and **(F)** other-limited metastasis (p=0.385, Figure 1F) after PSM. (The results of PSM were summarized in Table S4.)

We initially analyzed the effect of radiotherapy on rectal cancer patients with one site or organ and classified those into liver-limited, lung-limited and other-limited mRC. The results of the Cox regression model are displayed in Table S3. The multivariable Cox regression analysis confirmed that radiotherapy can be used as a prognostic factor for rectal liver-limited metastasis (p=0.007, Figure 4) but failed to improve survival for rectal lung-limited (p=0.060, Figure 4) and other-limited metastasis (p=0.596, Figure 4), which then was further confirmed by log-rank survival analysis after PSM. Table S4 displayed the characteristics of patients with M1a mRC before and after PSM. All of the three groups can obtain survival benefit from radiotherapy without the impact of PSM (p<0.001, Figure 5A-C). However, radiotherapy was able to improve OS of patients with rectal liver-limited metastasis (p=0.023, Figure 5D) and cannot provide survival benefit for rectal lung-limited (p=0.386, Figure 5E) and other-limited metastasis (p=0.385, Figure 5F).

### The effect of radiotherapy on M1b metastatic rectal cancer

**Figure 6:**
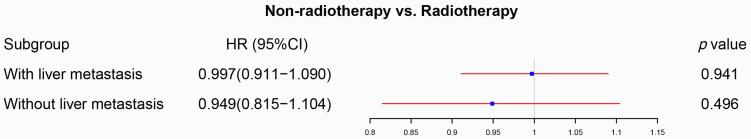
The forest plot illustrated the effect of radiotherapy on M1b rectal cancer. Radiotherapy was not able to significantly affect OS of M1b mRC patients with (p=0.941) and without liver metastasis (p=0.496). (The results were extracted from Table S5.)

**Figure 7:**
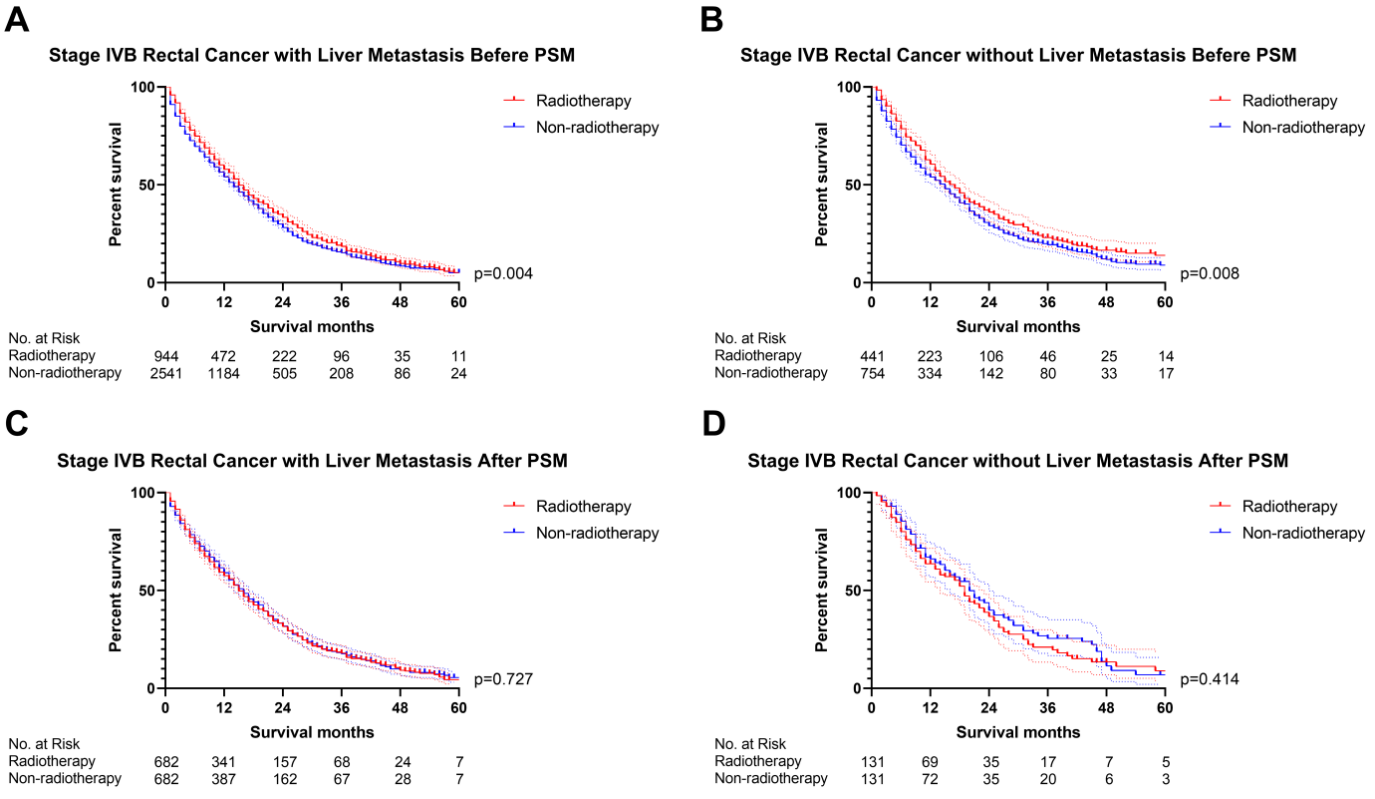
The survival curves indicated that **(A)** M1b rectal cancer with liver metastasis (p=0.004) and **(B)** M1b rectal cancer without liver metastasis (p=0.008) can obtain survival benefit from radiotherapy before PSM; However, radiotherapy cannot provide survival benefit for** (C)** M1b rectal cancer with liver metastasis (p=0.727) and **(D)** M1b rectal cancer without liver metastasis (p=0.414) after PSM. (The results of PSM were summarized in Table S6.)

The previous section proved that radiotherapy can only improve the prognosis of liver-limited mRC. Therefore, we divided the patients with M1b metastatic rectal cancer into ones with and without liver metastasis. Cox regression analysis (Table S5) confirmed that radiotherapy was not able to significantly affect OS of M1b mRC patients with (p=0.941, Figure 6) and without liver metastasis (p=0.496, Figure 6). The results of PSM were consistent with the Cox regression models (Before PSM: p=0.004 in M1b mRC patients with liver metastasis, Figure 7A; p=0.008 in M1b mRC patients without liver metastasis, Figure 7B; After PSM: p=0.727 in M1b mRC patients with liver metastasis, Figure 7C; p=0.414 in M1b mRC patients without liver metastasis, Figure 7D) (Table S6). Collectively, both of M1b mRC with and without liver metastasis cannot obtain survival benefit from radiotherapy.

## Discussion

To the best of our knowledge, this study was the first study to specifically investigate the effect of radiotherapy on metastatic rectal cancer patients based on the metastatic site. Somatic mutations and microsatellite instability status of the primary neoplasm have been previously indicated to impact patterns of colorectal metastasis 7, 12-14, which indicated that the molecular phenotype of the primary tumor is one of the key factors in determining the metastatic site in rectal cancer. Meanwhile, a large number of studies reported that the radiosensitivity of rectal cancer was related to the molecular phenotype 15-18. Hence, the metastatic site may be used as a factor in radiotherapy decisions for mRC.

Currently, radiotherapy is the recommended treatment option for patients with synchronous mRC according the National Comprehensive Cancer Network (NCCN) Clinical Practice Guidelines 19. A previous study using the SEER database demonstrated that radiotherapy was associated with a significant survival advantage in mRC without considering the metastatic site 8, which was consistent with our preliminary results. Actually, radiotherapy cannot provide survival benefit to mRC when the Cox regression analysis and PSM included the metastatic site. Therefore, the metastatic site may be an important factor affecting the sensitivity of mRC to radiotherapy. Moreover, a retrospective clinical study containing 89 synchronous rectal liver metastasis patients suggested that radiotherapy could significantly reduce the pelvic failure rate, but regrettably failed to explore the relationship between radiotherapy and overall survival 20. These preliminary evidences prompted us to further explore the role of radiotherapy on mRC according to the metastatic site. The final results confirmed that radiotherapy can only improve OS of patients with rectal liver-limited metastasis in this study.

The underlying mechanisms driving patterns of rectal metastasis are somewhat unclear. However, the metastatic site can be used as an indicator of the metastatic pattern of rectal cancer. Clinical evidence indicated that venous drainage of the colorectum into the portal system likely influences the pattern of metastatic spread first to the liver, and then to the lungs through the systemic circulation 21, 22. The high frequency of lung metastasis has been attributed to the potential hematogenous spread of distal rectal cancer through the inferior iliac veins and the inferior vena cava 22. Bone metastasis typically occurs via hematogenous dissemination 23. The metastasis pattern of rectal cancer with distant lymph node metastasis may be unique. Unfortunately, the SEER database does not provide detail information regarding distant lymph node metastasis. One feature of rectal liver-limited is that the metastatic pathway does not involve the systemic circulatory system, which, contrarily, is involved in the rectal metastasis to lung, bone, brain. Meanwhile, we explored the effect of radiotherapy on M1b mRC with liver metastasis, that metastatic mechanism may involve both of portal system and systemic circulation. Although the mechanism is unclear, radiotherapy may not provide survival benefits to mRC patients with metastatic pathway involving systemic circulation.

Our previous research explored the prognostic factors of colorectal liver-limited and lung-limited metastasis, that were inconsistent between the two groups 24, 25. Similarly, T staging can be used as a prognostic factor for rectal lung-limited metastasis but not for rectal liver-limited metastasis in this study (Table S3). On the contrary, N staging was associated with OS of rectal liver-limited metastasis and failed to affect OS of rectal lung-limited metastasis (Table S3). Moreover, both of T and N staging were not related to OS of rectal other-limited metastasis in the multivariable Cox regression analysis. These results reflected, to some extent, that the different metastasis sites were caused by nonidentical metastasis pathways in rectal cancer, which need to be blocked by distinct treatments. Altogether, effective treatment methods need to be explored to improve the prognosis of mRC by targeted blocking the metastatic pathways.

This study taking advantage of the large patient population of the SEER database is able to provide credible evidence regarding radiotherapy options and promote individualized treatment for mRC. First of all, our study can well encourage patients with rectal liver-limited to receive radiotherapy. Oncologists and patients did not realize the positive effect of radiotherapy on rectal liver-limited metastasis, which was the main reason for the disappointing low percentage of rectal liver-limited metastases patients receiving radiotherapy (32.04% in this study). In addition, this research can prompt oncologists to explore unique treatment strategies suitable for rectal cancer with different metastatic sites. In fact, current treatment strategies for mRC are mostly based on the experiences from rectal liver metastases 26-28. However, such strategies may not improve the prognosis of all mRC patients, such as the role of radiotherapy in this study. Meanwhile, the different results between with and without the metastatic site as an analysis factor reminded rectal cancer scholars that the metastatic site cannot be ignored in the research of metastatic rectal cancer. However, we still need to explore the molecular mechanism regarding that rectal liver-limited metastasis can benefit from radiotherapy, and radiotherapy resistance in patients with rectal cancer metastasis to other sites, which is of great significance for us to understand the molecular mechanism of rectal cancer radiotherapy sensitivity. The exploration of these molecular mechanisms is also helpful for us to predict the most likely metastasis sites of locoregional rectal cancer, so as to formulate targeted treatment strategies.

This study has certain limitations. As a non-random retrospective study, selection bias and confounding factors inevitably existed in this study. Even though PSM analysis was used in this study to remedy these defects, there were still some unrecognized confounders and some known confounders that could not be controlled. For example, the degree of tumor invasion, the distance from the tumor to the anus, surgical complications and recovery will affect the decision-making of radiotherapy. However, these variables are currently not directly available from the SEER database, so they can only be controlled indirectly. In addition, we did not deeply discuss the effect of radiotherapy on rectal bone and brain metastasis due to the limitation of the number of cases. And we failed to analysis mRC with distant lymph node metastasis since the SEER database only recorded four sites of metastasis at diagnosis. Moreover, SEER database lacks some important data, such as ECOG score, surgical details (resectable status, surgical margin), chemotherapy (whether 5-FU based) and radiotherapy details (target design, technology and dose), which is undoubtedly one of the shortcomings of this study. Now with the advent of precision therapy, genomic data also have great clinical reference value for guiding prognosis and treatment, but this is not recorded in the SEER database. These missing variables are critical to prognosis and need to be discussed in future studies. At last, this study only accessed retrospective data and need to be further verified by prospective research in the future.

## Conclusion

The metastatic site might serve for radiotherapy-decision in patients with synchronous metastatic rectal cancer. Radiotherapy appears to improve overall survival only in patients with rectal liver-limited metastasis. These findings are likely to inform rectal oncologists in counseling patients with stage IV rectal cancer with synchronous metastatic disease seeking prognostic information when weighing radiotherapy decisions.

## Supplementary Material

Supplementary tables.

## Figures and Tables

**Table 1:** Characteristics of metastatic rectal cancer

Characteristics	Total (n=10407)		Non-radiotherapy (n=6867)		Radiotherapy (n=3540)		*p*-value
	N	%	N	%	N	%	
**Insurance**							0.693
Yes	9708	93.28%	6401	93.21%	3307	93.42%	
No/NOS	699	6.72%	466	6.79%	233	6.58%	
**Gender**							0.651
Female	4003	38.46%	2652	38.62%	1351	38.16%	
Male	6404	61.54%	4215	61.38%	2189	61.84%	
**Age (years)**							<0.001
≤ 65	6578	63.21%	4166	60.67%	2412	68.14%	
> 65	3829	36.79%	2701	39.33%	1128	31.86%	
**Marital status**							0.001
Married	5237	50.32%	3378	49.19%	1859	52.51%	
Unmarried/NOS	5170	49.68%	3489	50.81%	1681	47.49%	
**Race**							0.394
White	8170	78.50%	5374	78.26%	2796	78.98%	
Non-white	2237	21.50%	1493	21.74%	744	21.02%	
**Pathologic grade**							0.004
Grade I/II	7397	71.08%	4161	60.59%	2236	63.16%	
Grade III/IV	1694	16.28%	1122	16.34%	572	16.16%	
Unknown	2316	22.25%	1584	23.07%	732	20.68%	
**Histologic type**							0.956
Adenocarcinomas	9829	94.45%	6485	94.44%	3344	94.46%	
MCC/SRCC	578	5.55%	382	5.56%	196	5.54%	
**T staging**							<0.001
T1-2	1487	14.29%	1053	15.33%	434	12.26%	
T3-4	5138	49.37%	3068	44.68%	2070	58.47%	
Tx	3782	36.34%	2746	39.99%	1036	29.27%	
**N staging**							0.660
N0	3599	34.58%	2501	36.42%	1098	31.02%	
N+	5630	54.10%	3476	50.62%	2154	60.85%	
Nx	1178	11.32%	890	12.96%	288	8.14%	
**Surgery**							<0.001
Standard Proctectomy	2744	26.37%	1786	26.01%	958	27.06%	
Simplified Proctectomy	1037	9.96%	529	7.70%	508	14.35%	
Non-proctectomy	6626	63.67%	4552	66.29%	2074	58.59%	
**Chemotherapy**							<0.001
Yes	8175	78.55%	4980	72.52%	3195	90.25%	
No	2232	21.45%	1887	27.48%	345	9.75%	
**CEA**							<0.001
Negative	1372	13.18%	785	11.43%	587	16.58%	
Positive	6245	60.01%	4210	61.31%	2035	57.49%	
NOS	2790	26.81%	1872	27.26%	918	25.93%	
**Metastatic site**							0.129
M1a: Liver-limited	4061	39.02%	2760	40.19%	1301	36.75%	
M1a: Lung-limited	850	8.17%	460	6.70%	390	11.02%	
M1a: Other-limited	816	7.84%	352	5.13%	464	13.11%	
M1b with liver metastasis	3485	33.49%	2541	37.00%	944	26.67%	
M1b without liver metastasis	1195	11.48%	754	10.98%	441	12.46%	

MCC: mucinous cell carcinoma; SRCC: signet ring cell carcinoma; NOS: Not otherwise specified.
