# Supplementary material for: The survival impact of radiotherapy on synchronous metastatic rectal cancer: metastatic site can serve for radiotherapy-decision
Source: J Cancer. 2022 Apr 4;13(7):2171–8. doi: 10.7150/jca.70894 (PMC9066223; doi:10.7150/jca.70894)
Supplement: Supplementary file 1 — Supplementary tables. [file jcav13p2171s1.pdf]

Table S1 Univariable and multivariable Cox regression model in total patients

Characteristics	Univariable analysis				Multivariable analysis with metastatic site				Multivariable analysis without metastatic site			
	HR	95% CI lower	95% CI upper	*p*-value	HR	95% CI lower	95% CI upper	*p*-value	HR	95% CI lower	95% CI upper	*p*-value
**Radiotherapy**				**<0.001**				**0.057**				**<0.001**
**Yes**		**reference**				**reference**				**reference**		
**No**	**1.397**	**1.327**	**1.471**	**<0.001**	**1.054**	**0.999**	**1.113**	**0.057**	**1.117**	**1.059**	**1.179**	**<0.001**
Insurance				0.053								
Yes		reference				NA				NA		
No/NOS	1.096	0.999	1.203	0.053								
Gender				0.138								
Female		reference				NA				NA		
Male	1.038	0.988	1.090	0.138								
Age(years)				<0.001				<0.001				<0.001
≤ 65		reference				reference				reference		
> 65	1.591	1.516	1.670	<0.001	1.386	1.318	1.458	<0.001	1.346	1.280	1.415	<0.001
Marital status				<0.001				<0.001				<0.001
Married		reference				reference				reference		
Unmarried/NOS	1.320	1.258	1.384	<0.001	1.155	1.100	1.212	<0.001	1.154	1.100	1.212	<0.001
Race				<0.001				0.014				0.020
White		reference				reference				reference		
Non-white	1.112	1.050	1.178	<0.001	1.075	1.015	1.139	0.014	1.071	1.011	1.135	0.020
Pathologic grade				<0.001				<0.001				<0.001
Grade I/II		reference				reference				reference		

Grade III/IV	1.527	1.432	1.628	<0.001	1.642	1.539	1.753	<0.001	1.664	1.560	1.776	<0.001
Unknown	1.497	1.412	1.587	<0.001	1.132	1.066	1.203	<0.001	1.139	1.072	1.209	<0.001
Histologic type				<0.001				<0.001				<0.001
Adenocarcinomas		reference				reference				reference		
MCC/SRCC	1.200	1.086	1.326	<0.001	1.222	1.102	1.355	<0.001	1.261	1.140	1.395	<0.001
T staging				<0.001				0.898				0.393
T1-2		reference				reference				reference		
T3-4	0.743	0.694	0.795	<0.001	1.017	0.945	1.094	0.650	1.017	0.946	1.094	0.646
Tx	1.228	1.141	1.321	<0.001	1.014	0.941	1.093	0.716	1.050	0.975	1.132	0.198
N staging				<0.001				<0.001				<0.001
N0		reference				reference				reference		
N+	0.791	0.751	0.834	<0.001	1.036	0.979	1.097	0.214	1.076	1.017	1.139	0.011
Nx	1.505	1.394	1.624	<0.001	1.217	1.124	1.317	<0.001	1.245	1.150	1.347	<0.001
Surgery				<0.001				<0.001				<0.001
Standard Proctectomy		reference				reference				reference		
Simplified Proctectomy	1.214	1.103	1.337	<0.001	1.209	1.097	1.333	<0.001	1.228	1.114	1.353	<0.001
Non-proctectomy	2.499	2.351	2.657	<0.001	2.137	1.991	2.294	<0.001	2.296	2.141	2.462	<0.001
Chemotherapy				<0.001				<0.001				<0.001
Yes		reference				reference				reference		
No	2.880	2.729	3.040	<0.001	2.617	2.470	2.773	<0.001	2.545	2.402	2.696	<0.001
CEA				<0.001				<0.001				<0.001
Negative		reference				reference				reference		
Positive	1.668	1.540	1.806	<0.001	1.427	1.316	1.548	<0.001	1.505	1.388	1.631	<0.001
NOS	1.584	1.453	1.728	<0.001	1.241	1.137	1.355	<0.001	1.283	1.176	1.400	<0.001
Metastatic site				<0.001				<0.001				

M1a: Liver-limited		reference				reference			Not included			
M1a: Lung-limited	0.905	0.820	1.000	0.049	0.752	0.680	0.832	<0.001				
M1a: Other-limited	0.807	0.728	0.894	<0.001	0.788	0.710	0.875	<0.001				
M1b with liver metastasis	1.726	1.633	1.825	<0.001	1.411	1.332	1.493	<0.001				
M1b without liver metastasis	1.515	1.400	1.641	<0.001	1.251	1.153	1.358	<0.001				
MCC: mucinous cell carcinoma; SRCC: signet ring cell carcinoma.												

Table S2 Characteristics of total patients after PSM with or without the metastatic site as matching factor

Characteristics	With the metastatic site					Without the metastatic site				
	Non-radiotherapy (n=1784)		Radiotherapy (n=1784)		*p*-value	Non-radiotherapy (n=2382)		Radiotherapy (n=2382)		*p*-value
	N	%	N	%		N	%	N	%	
Insurance					0.092					0.936
Yes	1716	96.19%	1734	97.20%		2302	96.64%	2303	96.68%	
No/NOS	68	3.81%	50	2.80%		80	3.36%	79	3.32%	
Gender					0.502					0.453
Female	616	34.53%	606	33.97%		889	37.32%	864	36.27%	
Male	1168	65.47%	1178	66.03%		1493	62.68%	1518	63.73%	
Age(years)					0.827					0.926
≤ 65	1240	69.51%	1246	69.84%		1611	67.63%	1614	67.76%	
> 65	544	30.49%	538	30.16%		771	32.37%	768	32.24%	
Marital status					0.867					0.977
Married	977	54.76%	972	54.48%		1267	53.19%	1266	53.15%	
Unmarried/NOS	807	45.24%	812	45.52%		1115	46.81%	1116	46.85%	
Race					0.555					0.275

White	1502	84.19%	1489	83.46%		1936	81.28%	1965	82.49%	
Non-white	282	15.81%	295	16.54%		446	18.72%	417	17.51%	
Pathologic grade					0.672					0.261
Grade I/II	1249	70.01%	1239	69.45%		1592	66.83%	1625	68.22%	
Grade III/IV	206	11.55%	206	11.55%		313	13.14%	309	12.97%	
Unknown	329	18.44%	339	19.00%		477	20.03%	448	18.81%	
Histologic type					0.291					0.924
Adenocarcinomas	1743	97.70%	1733	97.14%		2326	97.65%	2327	97.69%	
MCC/SRCC	41	2.30%	51	2.86%		56	2.35%	55	2.31%	
T staging					0.724					0.851
T1-2	208	11.66%	188	10.54%		255	10.71%	248	10.41%	
T3-4	1023	57.34%	1050	58.86%		1367	57.39%	1373	57.64%	
Tx	553	31.00%	546	30.61%		760	31.91%	761	31.95%	
N staging					0.465					0.821
N0	527	29.54%	535	29.99%		740	31.07%	735	30.86%	
N+	1109	62.16%	1118	62.67%		1462	61.38%	1463	61.42%	
Nx	148	8.30%	131	7.34%		180	7.56%	184	7.72%	
Surgery					0.710					0.961
Standard Proctectomy	530	29.71%	522	29.26%		692	29.05%	688	28.88%	
Simplified Proctectomy	142	7.96%	178	9.98%		179	7.51%	184	7.72%	
Non-proctectomy	1112	62.33%	1084	60.76%		1511	63.43%	1510	63.39%	
Chemotherapy					1.000					1.000
Yes	1630	91.37%	1630	91.37%		2153	90.39%	2153	90.39%	
No	154	8.63%	154	8.63%		229	9.61%	229	9.61%	
CEA					0.496					0.567

Negative	186	10.43%	199	11.15%		289	12.13%	293	12.30%	
Positive	1192	66.82%	1189	66.65%		1565	65.70%	1534	64.40%	
NOS	406	22.76%	196	10.99%		528	22.17%	555	23.30%	
Metastatic site					0.939					0.834
M1a: Liver-limited	836	46.86%	822	46.08%		1015	42.61%	895	37.57%	
M1a: Lung-limited	100	5.61%	111	6.22%		141	5.92%	246	10.33%	
M1a: Other-limited	80	4.48%	107	6.00%		104	4.37%	290	12.17%	
M1b with liver metastasis	636	35.65%	598	33.52%		899	37.74%	670	28.13%	
M1b without liver metastasis	132	7.40%	146	8.18%		223	9.36%	281	11.80%	
MCC: mucinous cell carcinoma; SRCC: signet ring cell carcinoma.										

Table S3 Univariable and multivariable Cox regression model in stage IVA rectal cancer patients

M1a: Liver-limited								
Characteristics	Univariable analysis				Multivariable analysis			
	HR	95% CI lower	95% CI upper	*p*-value	HR	95% CI lower	95% CI upper	*p*-value
**Radiotherapy**				**<0.001**				**0.007**
**Yes**		**reference**				**reference**		
**No**	**1.487**	**1.362**	**1.624**	**<0.001**	**1.135**	**1.036**	**1.244**	**0.007**
Insurance				.789				
Yes		reference				NA		
No/NOS	1.022	0.871	1.199	0.789				
Gender				0.437				
Female		reference				NA		
Male	1.034	0.950	1.125	0.437				

Age(years)				<0.001				<0.001
≤ 65		reference				reference		
> 65	1.733	1.599	1.879	<0.001	1.414	1.300	1.537	<0.001
Marital status				<0.001				<0.001
Married		reference				reference		
Unmarried/NOS	1.409	1.301	1.525	<0.001	1.242	1.147	1.345	<0.001
Race				0.044				0.377
White		reference				reference		
Non-white	1.105	1.003	1.217	0.044	1.045	0.948	1.153	0.377
Pathologic grade				<0.001				<0.001
Grade I/II		reference				reference		
Grade III/IV	1.454	1.302	1.623	<0.001	1.651	1.477	1.846	<0.001
Unknown	1.613	1.461	1.781	<0.001	1.150	1.037	1.274	0.008
Histologic type				0.999				
Adenocarcinomas		reference				reference		
MCC/SRCC	1<0.001	0.794	1.258	0.999				
T staging				<0.001				0.752
T1-2		reference				reference		
T3-4	0.687	0.616	0.766	<0.001	1.018	0.904	1.147	0.766
Tx	1.229	1.090	1.386	0.001	1.047	0.926	1.184	0.462
N staging				<0.001				0.012
N0		reference				reference		
N+	0.714	0.656	0.777	<0.001	1.016	0.924	1.117	0.743
Nx	1.481	1.301	1.686	<0.001	1.222	1.068	1.398	0.003
Surgery				<0.001				<0.001

Standard Proctectomy		reference				reference		
Simplified Proctectomy	1.166	1.006	1.351	0.042	1.148	0.989	1.334	0.070
Non-proctectomy	2.630	2.397	2.886	<0.001	2.264	2.029	2.527	<0.001
Chemotherapy				<0.001				<0.001
Yes		reference				reference		
No	3.258	2.973	3.571	<0.001	2.690	2.438	2.968	<0.001
CEA				<0.001				<0.001
Negative		reference				reference		
Positive	1.858	1.615	2.138	<0.001	1.483	1.287	1.710	<0.001
NOS	1.800	1.548	2.094	<0.001	1.310	1.123	1.527	0.001
M1a: Lung-limited								
Characteristics	Univariable analysis				Multivariable analysis			
	HR	95% CI lower	95% CI upper	*p*-value	HR	95% CI lower	95% CI upper	*p*-value
**Radiotherapy**				**<0.001**				**0.060**
**Yes**		**reference**				**reference**		
**No**	**1.400**	**1.165**	**1.683**	**<0.001**	**1.205**	**0.992**	**1.463**	**0.060**
Insurance				0.077				
Yes		reference				NA		
No/NOS	1.337	0.970	1.842	0.077				
Gender				0.532				
Female		reference				NA		
Male	1.060	0.883	1.272	0.532				
Age(years)				<0.001				<0.001
≤ 65		reference				reference		
> 65	1.870	1.556	2.247	<0.001	1.614	1.331	1.959	<0.001

Marital status				<0.001				0.072
Married		reference				reference		
Unmarried/NOS	1.417	1.179	1.703	<0.001	1.190	0.984	1.438	0.072
Race				0.281				
White		reference				NA		
Non-white	0.881	0.699	1.110	0.281				
Pathologic grade				0.010				<0.001
Grade I/II		reference				reference		
Grade III/IV	1.444	1.105	1.887	0.007	1.762	1.342	2.314	<0.001
Unknown	1.252	0.999	1.569	0.051	0.921	0.729	1.164	0.493
Histologic type				0.483				
Adenocarcinomas		reference				NA		
MCC/SRCC	1.166	0.760	1.790	0.483				
T staging				0.004				0.003
T1-2		reference				reference		
T3-4	0.949	0.732	1.230	0.691	1.636	1.231	2.173	0.001
Tx	1.394	1.027	1.892	0.033	1.345	0.982	1.842	0.065
N staging				<0.001				0.517
N0		reference				reference		
N+	0.696	0.574	0.844	<0.001	0.945	0.761	1.174	0.613
Nx	1.381	1.017	1.875	0.038	1.153	0.837	1.588	0.384
Surgery				<0.001				<0.001
Standard Proctectomy		reference				reference		
Simplified Proctectomy	1.114	0.776	1.600	0.559	1.265	0.875	1.828	0.211
Non-proctectomy	2.318	1.837	2.925	<0.001	2.489	1.900	3.260	<0.001

Chemotherapy				<0.001				<0.001
Yes		reference				reference		
No	2.654	2.184	3.226	<0.001	1.941	1.565	2.408	<0.001
CEA				0.118				
Negative		reference				NA		
Positive	1.284	1.006	1.639	0.045				
NOS	1.259	0.965	1.642	0.089				
M1a: Other-limited								
Characteristics	Univariable analysis				Multivariable analysis			
	HR	95% CI lower	95% CI upper	*p*-value	HR	95% CI lower	95% CI upper	*p*-value
**Radiotherapy**				**<0.001**				**0.596**
**Yes**		**reference**				**reference**		
**No**	**1.431**	**1.184**	**1.729**	**<0.001**	**1.060**	**0.855**	**1.313**	**0.596**
Insurance				0.094				
Yes		reference				NA		
No/NOS	0.738	0.517	1.053	0.094				
Gender				0.615				
Female		reference				NA		
Male	1.050	0.867	1.272	0.615				
Age(years)				<0.001				<0.001
≤ 65		reference				reference		
> 65	1.703	1.409	2.059	<0.001	1.497	1.229	1.824	<0.001
Marital status				0.015				0.300
Married		reference				reference		
Unmarried/NOS	1.265	1.046	1.529	0.015	1.109	0.912	1.349	0.300

Race				0.931				
White		reference				NA		
Non-white	1.010	0.802	1.272	0.931				
Pathologic grade				<0.001				<0.001
Grade I/II		reference				reference		
Grade III/IV	1.702	1.355	2.136	<0.001	1.757	1.393	2.215	<0.001
Unknown	1.515	1.175	1.953	0.001	1.178	0.903	1.539	0.228
Histologic type				0.914				
Adenocarcinomas		reference						
MCC/SRCC	0.982	0.704	1.370	0.914				
T staging				0.002				0.763
T1-2		reference				reference		
T3-4	0.874	0.649	1.177	0.376	1.119	0.817	1.533	0.482
Tx	1.364	0.961	1.934	0.082	1.123	0.782	1.612	0.530
N staging				<0.001				0.281
N0		reference				reference		
N+	0.824	0.671	1.011	0.063	1.121	0.895	1.406	0.320
Nx	1.845	1.264	2.695	0.002	1.348	0.906	2.006	0.141
Surgery				<0.001				<0.001
Standard Proctectomy		reference				reference		
Simplified Proctectomy	1.456	1.089	1.948	0.011	1.409	1.044	1.901	0.025
Non-proctectomy	2.418	1.938	3.017	<0.001	2.279	1.774	2.927	<0.001
Chemotherapy				<0.001				<0.001
Yes		reference				reference		
No	2.461	1.995	3.036	<0.001	2.418	1.896	3.084	<0.001

CEA				<0.001				<0.001
Negative		reference				reference		
Positive	1.864	1.414	2.458	<0.001	2.000	1.511	2.647	<0.001
NOS	1.706	1.276	2.279	<0.001	1.480	1.101	1.988	0.009
MCC: mucinous cell carcinoma; SRCC: signet ring cell carcinoma.								

Table S4 Characteristics of stage IVA rectal cancer patients before and after PSM

M1a: Liver-limited										
Characteristics	Before PSM					After PSM				
	Non-radiotherapy (n=2760)		Radiotherapy (n=1301)		*p*-value	Non-radiotherapy (n=926)		Radiotherapy (n=926)		*p*-value
	N	%	N	%		N	%	N	%	
Insurance					0.583					0.307
Yes	2592	93.91%	1216	93.47%		881	95.14%	890	96.11%	
No/NOS	168	6.09%	85	6.53%		45	4.86%	36	3.89%	
Gender					0.968					0.365
Female	914	33.12%	430	33.05%		294	31.75%	276	29.81%	
Male	1846	66.88%	871	66.95%		632	68.25%	650	70.19%	
Age(years)					<0.001					0.960
≤ 65	1720	62.32%	921	70.79%		643	69.44%	642	69.33%	
> 65	1040	37.68%	380	29.21%		283	30.56%	284	30.67%	
Marital status					0.002					0.347
Married	1416	51.30%	734	56.42%		522	56.37%	542	58.53%	
Unmarried/NOS	1344	48.70%	567	43.58%		404	43.63%	384	41.47%	
Race					0.437					0.360

White	2190	79.35%	1046	80.40%		756	81.64%	771	83.26%	
Non-white	570	20.65%	255	19.60%		170	18.36%	155	16.74%	
Pathologic grade					0.017					0.339
Grade I/II	1801	65.25%	898	69.02%		675	72.89%	662	71.49%	
Grade III/IV	400	14.49%	174	13.37%		111	11.99%	106	11.45%	
Unknown	559	20.25%	229	17.60%		140	15.12%	158	17.06%	
Histologic type					0.663					0.598
Adenocarcinomas	2679	97.07%	1266	97.31%		911	98.38%	908	98.06%	
MCC/SRCC	81	2.93%	35	2.69%		15	1.62%	18	1.94%	
T staging					0.006					0.306
T1-2	456	16.52%	160	12.30%		136	14.69%	110	11.88%	
T3-4	1294	46.88%	801	61.57%		552	59.61%	577	62.31%	
Tx	1010	36.59%	340	26.13%		238	25.70%	239	25.81%	
N staging					0.324					0.904
N0	1048	37.97%	414	31.82%		310	33.48%	297	32.07%	
N+	1390	50.36%	788	60.57%		541	58.42%	570	61.56%	
Nx	322	11.67%	99	7.61%		75	8.10%	59	6.37%	
Surgery					<0.001					0.158
Standard Proctectomy	930	33.70%	467	35.90%		352	38.01%	372	40.17%	
Simplified Proctectomy	226	8.19%	216	16.60%		94	10.15%	111	11.99%	
Non-proctectomy	1604	58.12%	618	47.50%		480	51.84%	443	47.84%	
Chemotherapy					<0.001					1.000
Yes	2081	75.40%	1213	93.24%		866	93.52%	866	93.52%	
No	679	24.60%	88	6.76%		60	6.48%	60	6.48%	
CEA					<0.001					0.593

Negative	281	10.18%	222	17.06%		118	12.74%	132	14.25%	
Positive	1730	62.68%	744	57.19%		579	62.53%	565	61.02%	
NOS	749	27.14%	335	25.75%		229	24.73%	229	24.73%	
M1a: Lung-limited										
Characteristics	Before PSM					After PSM				
	Non-radiotherapy (n=460)		Radiotherapy (n=390)		*p*-value	Non-radiotherapy (n=96)		Radiotherapy (n=96)		*p*-value
	N	%	N	%		N	%	N	%	
Insurance					0.083					0.702
Yes	433	94.13%	355	91.03%		93	96.88%	92	95.83%	
No/NOS	27	5.87%	35	8.97%		3	3.13%	4	4.17%	
Gender					0.552					1.000
Female	217	47.17%	176	45.13%		48	5	48	5	
Male	243	52.83%	214	54.87%		48	5	48	5	
Age(years)					0.007					0.770
≤ 65	218	47.39%	221	56.67%		56	58.33%	58	60.42%	
> 65	242	52.61%	169	43.33%		40	41.67%	38	39.58%	
Marital status					0.260					0.773
Married	211	45.87%	194	49.74%		54	56.25%	52	54.17%	
Unmarried/NOS	249	54.13%	196	50.26%		42	43.75%	44	45.83%	
Race					0.387					0.789
White	358	77.83%	313	80.26%		89	92.71%	88	91.67%	
Non-white	102	22.17%	77	19.74%		7	7.29%	8	8.33%	
Pathologic grade					0.363					0.619
Grade I/II	298	64.78%	263	67.44%		77	80.21%	76	79.17%	
Grade III/IV	56	12.17%	47	12.05%		7	7.29%	4	4.17%	

Unknown	106	23.04%	80	20.51%		12	12.50%	16	16.67%	
Histologic type					0.042					0.319
Adenocarcinomas	446	96.96%	367	94.10%		95	98.96%	96	10	
MCC/SRCC	14	3.04%	23	5.90%		1	1.04%	0		
T staging					0.688					0.806
T1-2	79	17.17%	40	10.26%		9	9.38%	11	11.46%	
T3-4	223	48.48%	250	64.10%		62	64.58%	60	62.50%	
Tx	158	34.35%	100	25.64%		25	26.04%	25	26.04%	
N staging					0.759					0.901
N0	189	41.09%	137	35.13%		32	33.33%	32	33.33%	
N+	219	47.61%	227	58.21%		57	59.38%	58	60.42%	
Nx	52	11.30%	26	6.67%		7	7.29%	6	6.25%	
Surgery					0.026					1.000
Standard Proctectomy	121	26.30%	106	27.18%		30	31.25%	28	29.17%	
Simplified Proctectomy	28	6.09%	69	17.69%		5	5.21%	9	9.38%	
Non-proctectomy	311	67.61%	215	55.13%		61	63.54%	59	61.46%	
Chemotherapy					<0.001					1.000
Yes	305	66.30%	349	89.49%		86	89.58%	86	89.58%	
No	155	33.70%	41	10.51%		10	10.42%	10	10.42%	
CEA					0.030					0.810
Negative	86	18.70%	89	22.82%		12	12.50%	15	15.63%	
Positive	227	49.35%	201	51.54%		63	65.63%	59	61.46%	
NOS	147	31.96%	100	25.64%		21	21.88%	22	22.92%	
M1a: Other-limited										
Characteristics	Before PSM					After PSM				

	Non-radiotherapy (n=352)		Radiotherapy (n=464)		*p*-value	Non-radiotherapy (n=85)		Radiotherapy (n=85)		*p*-value
	N	%	N	%		N	%	N	%	
Insurance					0.540					0.353
Yes	327	92.90%	436	93.97%		78	91.76%	81	95.29%	
No/NOS	25	7.10%	28	6.03%		7	8.24%	4	4.71%	
Gender					0.338					1.000
Female	159	45.17%	194	41.81%		33	38.82%	33	38.82%	
Male	193	54.83%	270	58.19%		52	61.18%	52	61.18%	
Age(years)					0.001					0.527
≤ 65	188	53.41%	303	65.30%		56	65.88%	52	61.18%	
> 65	164	46.59%	161	34.70%		29	34.12%	33	38.82%	
Marital status					<0.001					0.879
Married	148	42.05%	254	54.74%		44	51.76%	43	50.59%	
Unmarried/NOS	204	57.95%	210	45.26%		41	48.24%	42	49.41%	
Race					0.568					0.552
White	271	76.99%	365	78.66%		80	94.12%	78	91.76%	
Non-white	81	23.01%	99	21.34%		5	5.88%	7	8.24%	
Pathologic grade					0.473					0.735
Grade I/II	204	57.95%	291	62.72%		55	64.71%	60	70.59%	
Grade III/IV	88	25.00%	90	19.40%		22	25.88%	15	17.65%	
Unknown	60	17.05%	83	17.89%		8	9.41%	10	11.76%	
Histologic type					0.763					0.314
Adenocarcinomas	323	91.76%	423	91.16%		82	96.47%	84	98.82%	
MCC/SRCC	29	8.24%	41	8.84%		3	3.53%	1	1.18%	
T staging					0.389					0.877

T1-2	44	12.50%	45	9.70%		4	4.71%	4	4.71%	
T3-4	202	57.39%	309	66.59%		62	72.94%	61	71.76%	
Tx	106	30.11%	110	23.71%		19	22.35%	20	23.53%	
N staging					0.127					0.867
N0	125	35.51%	129	27.80%		14	16.47%	14	16.47%	
N+	204	57.95%	313	67.46%		66	77.65%	67	78.82%	
Nx	23	6.53%	22	4.74%		5	5.88%	4	4.71%	
Surgery					0.631					0.752
Standard Proctectomy	131	37.22%	169	36.42%		41	48.24%	38	44.71%	
Simplified Proctectomy	48	13.64%	85	18.32%		5	5.88%	7	8.24%	
Non-proctectomy	173	49.15%	210	45.26%		39	45.88%	40	47.06%	
Chemotherapy					<0.001					1.000
Yes	214	60.80%	427	92.03%		78	91.76%	78	91.76%	
No	138	39.20%	37	7.97%		7	8.24%	7	8.24%	
CEA					0.043					0.309
Negative	74	21.02%	103	22.20%		11	12.94%	15	17.65%	
Positive	147	41.76%	231	49.78%		43	50.59%	44	51.76%	
NOS	131	37.22%	130	28.02%		31	36.47%	26	30.59%	
MCC: mucinous cell carcinoma; SRCC: signet ring cell carcinoma.										

Table S5 Univariable and multivariable Cox regression model in stage IVB rectal cancer patients

M1b with liver metastasis		
Characteristics	Univariable analysis	Multivariable analysis

	HR	95% CI lower	95% CI upper	*p*-value	HR	95% CI lower	95% CI upper	*p*-value
**Radiotherapy**				**0.005**				**0.941**
**Yes**		**reference**				**reference**		
**No**	**1.133**	**1.038**	**1.237**	**0.005**	**0.997**	**0.911**	**1.090**	**0.941**
Insurance				0.437				
Yes		reference				NA		
No/NOS	1.060	0.916	1.226	0.437				
Gender				0.091				
Female		reference				NA		
Male	1.071	0.989	1.160	0.091				
Age(years)				<0.001				<0.001
≤ 65		reference				reference		
> 65	1.543	1.424	1.671	<0.001	1.339	1.233	1.453	<0.001
Marital status				<0.001				<0.001
Married		reference				reference		
Unmarried/NOS	1.263	1.169	1.365	<0.001	1.154	1.067	1.248	<0.001
Race				<0.001				0.003
White		reference				reference		
Non-white	1.198	1.093	1.312	<0.001	1.149	1.048	1.260	0.003
Pathologic grade				<0.001				<0.001
Grade I/II		reference				reference		
Grade III/IV	1.487	1.331	1.660	<0.001	1.469	1.314	1.643	<0.001
Unknown	1.304	1.191	1.427	<0.001	1.124	1.023	1.235	0.015
Histologic type				0.027				0.157
Adenocarcinomas		reference				reference		

MCC/SRCC	1.236	1.024	1.492	0.027	1.148	0.948	1.389	0.157
T staging				<0.001				0.530
T1-2		reference				reference		
T3-4	0.813	0.727	0.909	<0.001	0.937	0.833	1.055	0.286
Tx	1.118	0.998	1.253	0.054	0.944	0.840	1.061	0.334
N staging				<0.001				0.009
N0		reference				reference		
N+	0.904	0.830	0.986	0.022	1.055	0.963	1.156	0.252
Nx	1.387	1.232	1.560	<0.001	1.211	1.072	1.367	0.002
Surgery				<0.001				<0.001
Standard Proctectomy		reference				reference		
Simplified Proctectomy	1.144	0.944	1.386	0.170	1.143	0.942	1.386	0.174
Non-proctectomy	1.938	1.725	2.178	<0.001	1.860	1.633	2.119	<0.001
Chemotherapy				<0.001				<0.001
Yes		reference				reference		
No	2.874	2.633	3.136	<0.001	2.701	2.463	2.961	<0.001
CEA				<0.001				<0.001
Negative		reference				reference		
Positive	1.491	1.277	1.741	<0.001	1.417	1.213	1.655	<0.001
NOS	1.491	1.263	1.760	<0.001	1.253	1.061	1.479	0.008
M1b without liver metastasis								
Characteristics	Univariable analysis				Multivariable analysis			
	HR	95% CI lower	95% CI upper	*p*-value	HR	95% CI lower	95% CI upper	*p*-value
**Radiotherapy**				**0.010**				**0.536**
**Yes**		**reference**				**reference**		

**No**	**1.207**	**1.046**	**1.394**	**0.010**	**0.953**	**0.819**	**1.109**	**0.536**
Insurance				.753				
Yes		reference				NA		
No/NOS	1.048	0.783	1.403	0.753				
Gender				0.343				
Female		reference				NA		
Male	1.069	0.931	1.227	0.343				
Age(years)				<0.001				<0.001
≤ 65		reference				reference		
> 65	1.542	1.341	1.772	<0.001	1.307	1.130	1.513	<0.001
Marital status				0.222				
Married		reference				NA		
Unmarried/NOS	1.089	0.950	1.250	0.222				
Race				0.583				
White		reference				NA		
Non-white	1.047	0.889	1.233	0.583				
Pathologic grade				<0.001				<0.001
Grade I/II		reference				reference		
Grade III/IV	1.605	1.369	1.882	<0.001	1.964	1.663	2.318	<0.001
Unknown	1.387	1.163	1.653	<0.001	1.278	1.065	1.533	0.008
Histologic type				0.005				0.024
Adenocarcinomas		reference				reference		
MCC/SRCC	1.283	1.080	1.525	0.005	1.228	1.027	1.468	0.024
T staging				<0.001				0.855
T1-2		reference				reference		

T3-4	0.758	0.617	0.933	0.009	1.064	0.854	1.325	0.580
Tx	1.047	0.837	1.309	0.687	1.039	0.825	1.308	0.746
N staging				<0.001				0.156
N0		reference				reference		
N+	0.764	0.655	0.890	0.001	1.018	0.863	1.201	0.834
Nx	1.342	1.079	1.669	0.008	1.244	0.989	1.564	0.062
Surgery				<0.001				<0.001
Standard Proctectomy		reference				reference		
Simplified Proctectomy	1.207	0.930	1.566	0.158	1.300	0.995	1.697	0.054
Non-proctectomy	2.053	1.719	2.452	<0.001	2.159	1.762	2.646	<0.001
Chemotherapy				<0.001				<0.001
Yes		reference				reference		
No	2.628	2.260	3.057	<0.001	2.652	2.249	3.127	<0.001
CEA				0.061				
Negative		reference				NA		
Positive	1.245	1.034	1.498	0.020				
NOS	1.220	0.993	1.499	0.059				
MCC: mucinous cell carcinoma; SRCC: signet ring cell carcinoma.								

Table S6 Characteristics of stage IVB rectal cancer before and after PSM

M1b with liver metastasis									
Characteristics	Before PSM					After PSM				
	Non-radiotherapy (n=2541)		Radiotherapy (n=944)		*p*-value	Non-radiotherapy (n=682)		Radiotherapy (n=682)		*p*-value
	N	%	N	%		N	%	N	%	

Insurance					0.097					0.801
Yes	2337	91.97%	884	93.64%		648	95.01%	650	95.31%	
No/NOS	204	8.03%	60	6.36%		34	4.99%	32	4.69%	
Gender					0.144					0.820
Female	1003	39.47%	347	36.76%		234	34.31%	238	34.90%	
Male	1528	60.13%	597	63.24%		448	65.69%	444	65.10%	
Age(years)					<0.001					0.436
≤ 65	1603	63.09%	663	70.23%		494	72.43%	481	70.53%	
> 65	938	36.91%	281	29.77%		188	27.57%	201	29.47%	
Marital status					0.531					0.957
Married	1224	48.17%	466	49.36%		344	50.44%	345	50.59%	
Unmarried/NOS	1317	51.83%	478	50.64%		338	49.56%	337	49.41%	
Race					0.456					0.052
White	1971	77.57%	721	76.38%		527	77.27%	556	81.52%	
Non-white	570	22.43%	223	23.62%		155	22.73%	126	18.48%	
Pathologic grade					0.273					0.801
Grade I/II	1495	58.84%	569	60.28%		420	61.58%	427	62.61%	
Grade III/IV	358	14.09%	140	14.83%		88	12.90%	82	12.02%	
Unknown	688	27.08%	235	24.89%		174	25.51%	173	25.37%	
Histologic type					0.917					0.880
Adenocarcinomas	2434	95.79%	905	95.87%		660	96.77%	659	96.63%	
MCC/SRCC	107	4.21%	39	4.13%		22	3.23%	23	3.37%	
T staging					0.001					0.809
T1-2	394	15.51%	131	13.88%		94	13.78%	75	11.00%	
T3-4	970	38.17%	473	50.11%		299	43.84%	343	50.29%	

Tx	1177	46.32%	340	36.02%		289	42.38%	264	38.71%	
N staging					0.992					0.789
N0	891	35.06%	286	30.30%		209	30.65%	201	29.47%	
N+	1263	49.70%	559	59.22%		400	58.65%	410	60.12%	
Nx	387	15.23%	99	10.49%		73	10.70%	71	10.41%	
Surgery					0.072					0.723
Standard Proctectomy	421	16.57%	123	13.03%		90	13.20%	86	12.61%	
Simplified Proctectomy	148	5.82%	74	7.84%		37	5.43%	36	5.28%	
Non-proctectomy	1972	77.61%	747	79.13%		555	81.38%	560	82.11%	
Chemotherapy					<0.001					0.872
Yes	1869	73.55%	816	86.44%		593	86.95%	595	87.24%	
No	672	26.45%	128	13.56%		89	13.05%	87	12.76%	
CEA					0.251					1.000
Negative	196	7.71%	83	8.79%		42	6.16%	47	6.89%	
Positive	1692	66.59%	631	66.84%		501	73.46%	491	71.99%	
NOS	653	25.70%	230	24.36%		139	20.38%	144	21.11%	
M1b without liver metastasis										
Characteristics	Before PSM					After PSM				
	Non-radiotherapy (n=754)		Radiotherapy (n=441)		*p*-value	Non-radiotherapy (n=131)		Radiotherapy (n=131)		*p*-value
	N	%	N	%		N	%	N	%	
Insurance					0.943					0.252
Yes	712	94.43%	416	94.33%		129	98.47%	126	96.18%	
No/NOS	42	5.57%	25	5.67%		2	1.53%	5	3.82%	
Gender					0.651					0.536
Female	359	47.61%	204	46.26%		61	46.56%	56	42.75%	

Male	395	52.39%	237	53.74%		70	53.44%	75	57.25%	
Age(years)					<0.001					0.416
≤ 65	437	57.96%	304	68.93%		96	73.28%	90	68.70%	
> 65	317	42.04%	137	31.07%		35	26.72%	41	31.30%	
Marital status					0.420					0.324
Married	379	50.27%	211	47.85%		72	54.96%	64	48.85%	
Unmarried/NOS	375	49.73%	230	52.15%		59	45.04%	67	51.15%	
Race					0.388					0.618
White	584	77.45%	351	79.59%		111	84.73%	108	82.44%	
Non-white	170	22.55%	90	20.41%		20	15.27%	23	17.56%	
Pathologic grade					0.914					0.196
Grade I/II	363	48.14%	215	48.75%		77	58.78%	70	53.44%	
Grade III/IV	220	29.18%	121	27.44%		31	23.66%	28	21.37%	
Unknown	171	22.68%	105	23.81%		23	17.56%	33	25.19%	
Histologic type					0.003					0.828
Adenocarcinomas	603	79.97%	383	86.85%		120	91.60%	119	90.84%	
MCC/SRCC	151	20.03%	58	13.15%		11	8.40%	12	9.16%	
T staging					0.028					0.919
T1-2	80	10.61%	58	13.15%		11	8.40%	11	8.40%	
T3-4	379	50.27%	237	53.74%		75	57.25%	74	56.49%	
Tx	295	39.12%	146	33.11%		45	34.35%	46	35.11%	
N staging					0.680					0.910
N0	248	32.89%	132	29.93%		32	24.43%	32	24.43%	
N+	400	53.05%	267	60.54%		89	67.94%	88	67.18%	
Nx	106	14.06%	42	9.52%		10	7.63%	11	8.40%	

Surgery					0.644					0.389
Standard Proctectomy	183	24.27%	93	21.09%		34	25.95%	30	22.90%	
Simplified Proctectomy	79	10.48%	64	14.51%		13	9.92%	9	6.87%	
Non-proctectomy	492	65.25%	284	64.40%		84	64.12%	92	70.23%	
Chemotherapy					<0.001					1.000
Yes	511	67.77%	390	88.44%		117	89.31%	117	89.31%	
No	243	32.23%	51	11.56%		14	10.69%	14	10.69%	
CEA					0.685					0.337
Negative	148	19.63%	90	20.41%		27	20.61%	21	16.03%	
Positive	414	54.91%	228	51.70%		76	58.02%	78	59.54%	
NOS	192	25.46%	123	27.89%		28	21.37%	32	24.43%	
MCC: mucinous cell carcinoma; SRCC: signet ring cell carcinoma.										
